# Influence of environmental, economic, dietary, and behavioural factors on high myopia onset in children: a 4-year large-scale cohort study

**DOI:** 10.7189/jogh.16.04298

**Published:** 2026-10-02

**Authors:** Zixun Wang, Jingtao Yu, Desheng Song, Jinghui Wang, Hua Yan, Bei Du, Ruihua Wei

**Affiliations:** 1 Tianjin Key Laboratory of Retinal Functions and Diseases, Tianjin Branch of National Clinical Research Center for Ocular Disease, Eye Institute and School of Optometry, Tianjin Medical University Eye Hospital, Tianjin, China; 2 TMUE Optometric Technology Co., Ltd., Tianjin, China; 3 Tianjin Key Laboratory of Ocular Trauma, Tianjin, China; 4 Department of Ophthalmology, Tianjin Medical University General Hospital, Laboratory of Molecular Ophthalmology, Tianjin Medical University, Tianjin, China

**Keywords:** high myopia, childhood myopia, socioeconomic status, neighbourhood greenness, outdoor activity, dietary patterns

## Abstract

**Background:**

High myopia (HM) has become a major global public health concern, leading to irreversible vision loss and a growing socioeconomic burden. Despite rapid urbanisation and education-driven lifestyle changes, few large-scale longitudinal studies have examined how social and environmental factors interact with children’s daily behaviours to influence the progression from myopia to HM.

**Methods:**

This 4-year population-based cohort study (2021–2024) included 204,238 students (grades 1–9) with baseline myopia from the TCARE programme. Exposures included area-level socioeconomic status (SES), school-based greenness (1 km normalised difference vegetation index (NDVI)), and behavioural factors (daily outdoor activity > two *vs.*≤2 hours and three dietary patterns: energy-dense, plant-based, and traditional nutrient-rich).

**Results:**

Among 204,238 participants (mean age 10.8 ± 4.7 years), 26,879 (13.2%) developed HMs. Children living in low-SES areas had a 54% greater risk than those living in high-SES areas did (hazard ratio (HR) = 1.54; 95% confidence interval (CI) = 1.49–1.59). Protective factors included outdoor activity >2 hours/d (HR = 0.92; 95% CI = 0.90–0.95) and plant-based diets (HR = 0.97; 95% CI = 0.94–1.00). A higher NDVI was associated with a generally lower risk across outdoor activity levels (HR = 0.90 *vs.* 0.94 in low-NDVI areas, *P* = 0.01), whereas low SES was associated with a weaker protective association of outdoor activity (HR = 0.96 *vs.* 0.89 in high-SES areas, *P* = 0.03). The strongest protective association was observed among children aged 6–12 years (HR = 0.88; 95% CI = 0.82–0.95).

**Conclusions:**

High myopia progression among children is driven primarily by socioeconomic inequities, whereas environmental greenness and outdoor behaviour provide measurable protective effects. Integrating SES-targeted resource allocation, urban greening, and early-life behavioural interventions is crucial for mitigating the paediatric HM epidemic.

Globally, uncorrected refractive error is the leading cause of visual impairment, affecting over two billion people, and it is often detected late [1]. Early prediction and intervention in children and adolescents are crucial for slowing myopia progression, improving visual outcomes and quality of life, and reducing socioeconomic burdens [2,3]. In China, projections indicate an overall myopia prevalence of 61.3% and a high myopia (HM) prevalence of 17.6% by 2050 [3]. High myopia, which often progresses to pathologic myopia (PM), carries significant risks of serious vision-threatening complications. Earlier onset and greater baseline refractive error increase these risks [4–6]. While adult PM cohorts exist, extensive longitudinal studies tracking school-age children from myopia onset to HM are lacking.

Myopia progression to HM is multifactorial. Macrolevel influences include genetics, the environment, and societal factors [5,7]. Our previous Tianjin Child and Adolescent Research on Eye (TCARE) study suggested that exposure to green space has protective effects on myopia development in schoolchildren [6]. However, this cross-sectional finding requires temporal validation, and HM was not the study endpoint.

Although myopia control options (functional eyewear, low-dose atropine) have multiplied, financial burdens and access disparities linked to socioeconomic status (SES) are significant [8–11]. A region's SES encompasses critical living conditions, education, and healthcare [12]. Sole reliance on metrics such as gross national product (GDP) fails to capture the holistic development that affects myopia progression to HM; factors such as economic stratification, medical investment, educational resources, and the environment require comprehensive assessment [13].

Behavioural management is a key prevention strategy. The International Myopia Institute (IMI) guidelines identify outdoor activity as protective and near work as potentially progressive [6,14]. Links between behavioural factors and myopia onset have been reported [15], but their relationship with HM progression remains unclear, as does the influence of diet on children progressing to HM [15]. Environmental and behavioural exposures may influence axial elongation through several biological pathways [16]. Increased exposure to outdoor light stimulates retinal dopamine release, thereby inhibiting excessive ocular growth [17]. Reduced accommodative demand and improved depth of focus conditions outdoors may further decrease hyperopic defocus-driven elongation [18]. Socioeconomic environments may also indirectly influence ocular growth through educational attainment, stress, sleep patterns, and access to preventive care [19].

Emerging evidence suggests that dietary factors may influence ocular growth through metabolic and structural pathways [20]. High glycaemic load diets may increase insulin and insulin-like growth factor signalling, which have been hypothesised to affect scleral extracellular matrix remodelling and axial elongation. Conversely, nutrient rich or plant based dietary patterns may provide antioxidants and micronutrients that support the integrity of retinal and scleral tissues [21]. These biological considerations provide a rationale for examining dietary patterns alongside environmental and behavioural exposures in the progression toward HM.

Therefore, this four years’ longitudinal study aims to investigate the impact of genetic, environmental, and socioeconomic factors, as well as individual behaviours, on progression to HM in non-HM children across diverse regions of Tianjin.

## METHODS

### Ethics approval and consent to participate

Ethical approval for this research was granted by the Ethics Committee of Tianjin Medical University Eye Hospital (KY-202467). All procedures adhered to the principles of the Declaration of Helsinki. Written informed consent was secured from each participant and their legal guardians prior to undergoing physical and ophthalmic examinations. Participation was entirely voluntary, with the option to discontinue at any stage without penalty.

The TCARE project is a large, population-based longitudinal cohort in Tianjin, China, built upon the city’s long-standing myopia surveillance system. This initiative implements semester-based monitoring during primary school years and annual follow-up assessments in secondary schools. Participating students are registered in individualised electronic health record systems that enable secure access to screening outcomes *via* smartphone-authenticated WeChat portals for both students and their guardians.

### Participants and procedures

Over the four years of 2021–2024 (eight follow-ups), we included students in grades 1–9 with myopia SE>−6D, more than one visit, available behavioural information as a baseline inclusion, and a follow-up period of at least one year. Exclusion criteria included individuals with prior cataract surgery, laser refractive surgery, orthokeratology, or active ocular inflammation. Based on the previously described inclusion criteria, we included 215,738 students in 2021 (**Figure 1**). After reviewing the exclusion criteria, 204,238 students were included in this analytical study. The diagnostic thresholds for myopia classification were mild (−3.00D<SE≤−0.50D), moderate (−6.00D<SE≤−3.00D), and HM (SE≤−6.00D), contingent on concurrent uncorrected visual acuity (UCVA)<5.0 [22]. Specific ocular and refractive examinations (UCVA and SE) are described in Section 1 in the **Online Supplementary Document**, and specific procedures have been detailed in our previous team studies [23].

**Figure 1:**
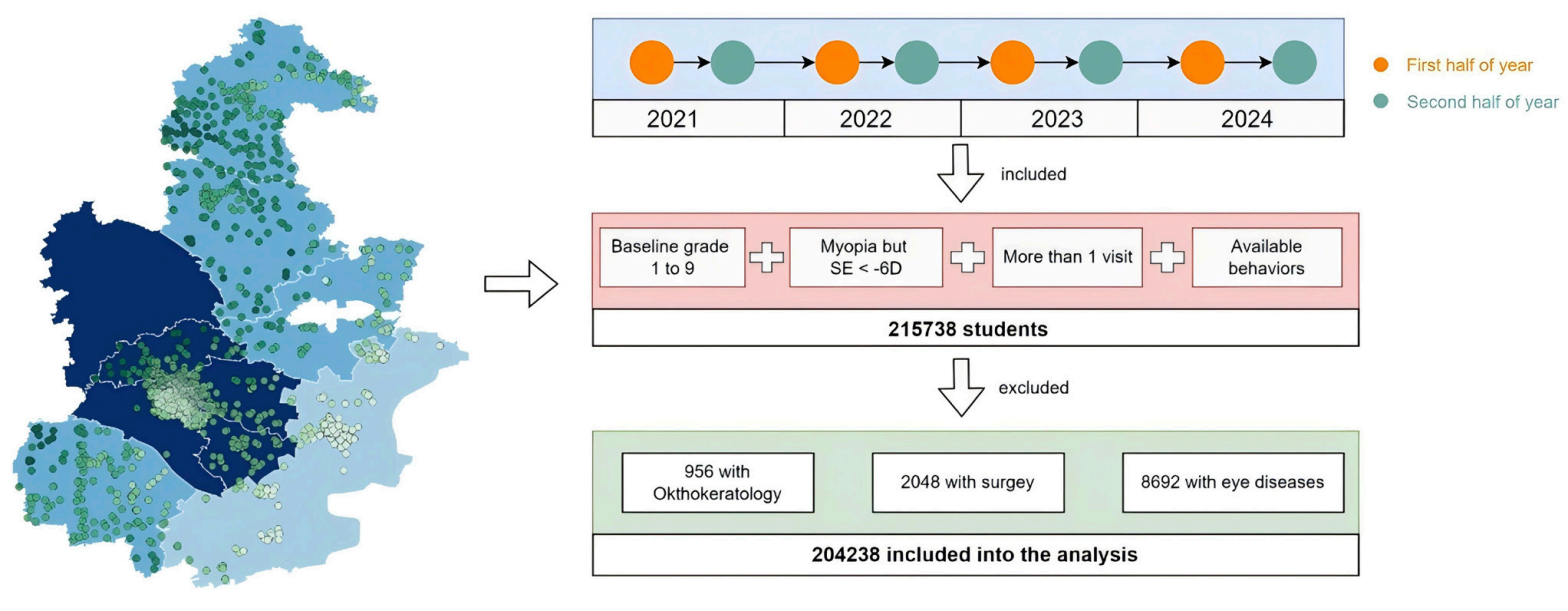
Study design and participant flow. Geographic distribution of participating student schools across all districts in Tianjin. Each dot represents one school, with colour intensity corresponding to the annual average Normalized Difference Vegetation Index (NDVI) within a 3-km buffer zone around each school; background shading denotes regional socioeconomic status (SES). Longitudinal follow-up timeline spanning 2021 to 2024, with bi-annual vision screening assessments. A total of 215,738 students fulfilled preliminary eligibility criteria (Grades 1–9 at baseline, spherical equivalent>−6 D consistent with high myopia, ≥2 clinical visits, and complete behavioural covariate data). After sequential exclusion of participants using orthokeratology lenses (n = 56), those with prior ocular surgery (n = 2,948), and individuals with other pre-existing ocular diseases (n = 8,692), the final analytical sample comprised 204,238 students. NDVI – Normalized Difference Vegetation Index, SES – socioeconomic status.

### Family history, behavioural, and dietary surveys

Demographic profiling (including age, sex, and educational status) was conducted by teaching staff during the prescreening phases. A population-based behavioural assessment was conducted using the TCARE instrument, administered *via* WeChat-based electronic surveys to a cross-sectional cohort randomly selected across eight Tianjin districts. This standardised survey included pedigree analysis of myopia predisposition and a comprehensive evaluation of paediatric behavioural patterns (Table S2 in the **Online Supplementary Document**). Behavioural and dietary variables were primarily collected at baseline and analysed as baseline exposures in longitudinal models. The surveys were conducted using stratified random sampling across participating districts to ensure demographic representativeness relative to the overall cohort population. Based on the questionnaire content, we classified the diets into three dietary patterns using factor analysis: an energy-dense pattern, a plant-based pattern, and a traditional nutrient-rich pattern (Figure S1 in the **Online Supplementary Document**). Outdoor activity was categorised as ≤2 hours/d and >2 hours/d based on recommendations from the International Myopia Institute and previous epidemiological evidence suggesting that approximately two hours of daily outdoor exposure represents a protective threshold for myopia prevention.

### Area-level SES

A multidimensional SES was developed to quantify regional development patterns, incorporating metrics of economic productivity, education investment, and healthcare resources. The index operationalises five core parameters: GDP per capita, the proportion of GDP allocated to education budgets, regional tertiary GDP per capita, the share of GDP earmarked for health systems, healthcare personnel density (physicians and nurses per 1000 people), and hospital bed availability per 1000 residents. All variables were derived from the Tianjin Municipal Government's authoritative statistical data for 2020. The data set underwent Z-normalisation before principal component extraction, formalised as:



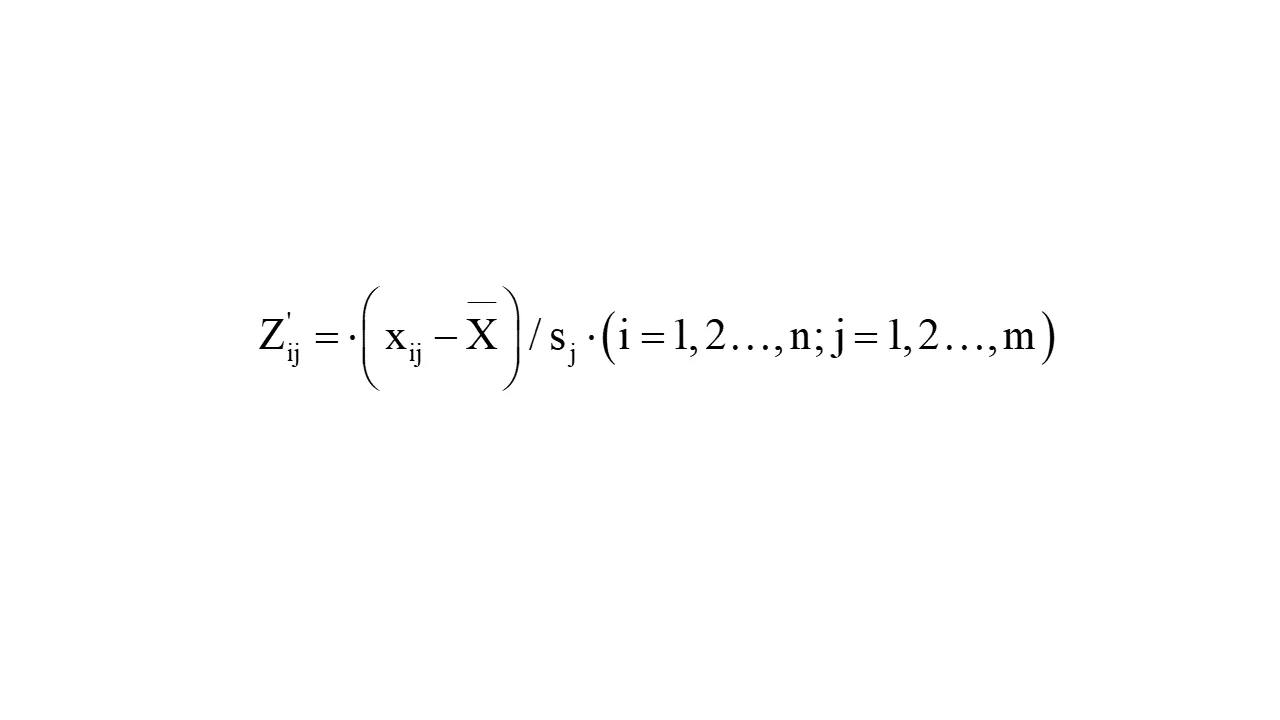



where indices i (1≤i ≤ n) and j (1≤j ≤ m) represent observational units and variables, respectively. Feature reduction *via* PCA yielded variance-maximising coefficients c_1_…cₚ, enabling the construction of an aggregate metric:

f = c1Z1 + c2Z2 + ⋯ + cpZp

Tianjin’s 16 administrative units were subsequently stratified into socioeconomic tertiles (high, medium, and low SES) based on quantile rankings of synthesised f scores. (Tables S3–4 in the **Online Supplementary Document**) The SESs in different areas of Tianjin were classified into three classes, low SES, medium SES, and high SES, based on the percentage of principal components. The specific SES classification of gravelly soil is shown in Figure S2 in the **Online Supplementary Document**.

### Green space

Vegetation quantification within school premises was conducted *via* MODIS-derived NDVI metrics. NDVI values were standardised prior to modelling to facilitate interpretation. This standardised satellite-derived vegetation index ((range: −1 to +1)) reflects vegetation density through normalised reflectance differences in the near-infrared (NIR) and visible red bands, with ascending values corresponding to increased plant coverage. Imaging data were acquired from NASA's Earth Observing System satellites (Terra and Aqua platforms), which feature MODIS-collected spectral bands critical for vegetation monitoring. Preprocessing involved sequential radiometric normalisation, atmospheric compensation, and geometric rectification *via* standardised planetary reference coefficients, followed by index computation. NDVI computation followed radiometric normalisation, atmospheric adjustment, and geometric correction (planetary reference coefficients), utilising the following formula:

NDVI = (NIR − RED) / (NIR + RED)

NIR and RED correspond to the near-infrared and visible-red spectral ranges, respectively [24].

Aquatic features (−1 to 0 NDVI range) were systematically excluded through null value assignment. Geospatial analysis incorporated school coordinates from Tianjin's educational registry and calculated 16 days’ composite NDVI means within concentric circular buffers (250 m, 500 m, and 1000 m radii) around each institute. Urban accessibility considerations defined these zones, with a 1000 m radius reflecting China's typical 15-minute pedestrian catchment. A triennial NDVI aggregate (2021) was generated to mitigate seasonal fluctuations and extreme events, thereby improving the quantification of longitudinal vegetation coverage.

### Outcomes

Myopia was defined as an equivalent SE≤−0.50 D [25], and HM was defined as an equivalent SE≤−6.00 D. SE was calculated by summing half of the spherical and half of the cylindrical eye. The outcome metrics for our cohort were primarily children with myopia who progressed from myopia to HM among students who were followed for more than one year.

### Statistical analysis

Continuous variables were quantified as mean ± standard deviation (μ ± σ), whereas categorical data were presented as frequency distributions with proportional percentages. Intergroup differences across these population descriptors were statistically evaluated using two-sample parametric testing (Student’s *t* test) and nonparametric Pearson χ^2^ contingency analysis. We applied a generalised linear mixed model to assess how high myopia relates to area-level socioeconomic status, outdoor activity, and school-based green space. This approach was selected because it accommodates both fixed and random components, allowing for the inclusion of interaction terms and thereby improving model interpretation and prediction. In this process, we perform multifactor adjustment and conduct interaction analysis. In addition, we analysed the population into different subgroups based on SES, NDVI, outdoor exercise time, and dietary patterns, and assessed the risk associated with baseline influences in these subgroups. Finally, we performed sensitivity analyses to assess the reliability of our findings, using logistic and linear regression analyses and adjusting for potential confounders. Missing data (<0.8%) showed no systematic association with demographic or outcome variables, suggesting approximate missing-at-random conditions; therefore, imputation was not performed. Analytical computations were performed in the *R,* version 4.2.2 (R Foundation, Vienna, Austria). The hypothesis evaluations maintained a two-tailed α threshold of 0.05.

## RESULTS

### Basic information and multifactor regression analysis

**Table 1** shows the baseline characteristics of myopia patients who progressed to HM. The study included 204,238 students; 26,879 (13.2%) developed incident HM during follow-up. **Table 1** presents baseline characteristics stratified by HM status. Significant differences were observed between groups in most variables (*P* < 0.005, except sex and average NDVI). Although the overall mean NDVI appeared narrowly distributed, the tertile-based categorisation revealed meaningful spatial contrasts across districts. The students who developed HMs were older at baseline (11.4 ± 4.4 *vs.* 10.6 ± 4.8 years, *P* < 0.001) and had worse baseline (SE = −4.1 ± 1.3D *vs.* −2.1 ± 1.2D, *P* < 0.001). A family history of myopia was more prevalent in the HM group (54.0% *vs.* 41.4%, *P* < 0.001), and these students had lower rates of daily outdoor activity >2 hours (13.5% *vs.* 15.3%, *P* < 0.001).

**Table 1:** Baseline characteristics of students by incident high myopia

Characteristic	Overall (n = 204,238)	No incident high myopia (n = 177,359)	Incident high myopia (n = 26,879)	*P*-value†
Baseline school age, mean (SD)*	10.7 (4.8)	10.6 (4.8)	11.4 (4.4)	<0.001
Baseline SER, mean (SD)*	−2.4 (1.4)	−2.1 (1.2)	−4.1 (1.3)	<0.001
Sex, n (%)				0.515
*Boys*	101,467 (49.7)	88,163 (49.7)	13,304 (49.5)	
*Girls*	102,771 (50.3)	89,196 (50.3)	13,575 (50.5)	
Family history of myopia, n (%)				<0.001
*No*	116,302 (56.9)	103,933 (58.6)	12,369 (46.0)	
*Yes*	87,936 (43.1)	73,426 (41.4)	14,510 (54.0)	
Daily outdoor activity time, n (%)				<0.001
*0–2 h*	173,423 (84.9)	150,171 (84.7)	23,252 (86.5)	
*>2 h*	30,815 (15.1)	27,188 (15.3)	3,627 (13.5)	
Daily near-work reading time, n (%)				0.005
*0–2 h*	142,487 (69.8)	124,307 (70.1)	18,180 (67.6)	
*>2 h*	61,751 (30.2)	53,052 (29.9)	8,699 (32.4)	
Dietary pattern, n (%)				0.005
*Energy-dense pattern*	73,280 (35.9)	63,416 (35.8)	9,864 (36.7)	
*Plant-based pattern*	71,123 (34.8)	61,808 (34.8)	9,315 (34.7)	
*Traditional nutrient-rich pattern*	59,835 (29.3)	52,135 (29.4)	7,700 (28.6)	
Average NDVI, mean (SD)*	0.1 (0.0)	0.1 (0.0)	0.1 (0.0)	0.096
Socioeconomic status (SES), n (%)				<0.001
*High*	63,042 (30.9)	55,272 (31.2)	7,770 (28.9)	
*Medium*	90,562 (44.3)	78,369 (44.2)	12,193 (45.4)	
*Low*	50,634 (24.8)	43,718 (24.6)	6,916 (25.7)	

NDVI – Normalized Difference Vegetation Index, SER – spherical equivalent refraction, SES – socioeconomic status

*Mean (standard deviation) for continuous variables; number of participants (percentage) for categorical variables.

†Independent samples *t* test for continuous variables; Pearson χ^2^ test for categorical variables.

We used Cox proportional hazards regression to assess the associations of outdoor activity, dietary patterns, socioeconomic status, and the NDVI with the risk of myopia progression to HM (**Table 2**). The results revealed that the risk of myopia progression was significantly lower for those who spent ≥2 hours per day outdoors (HR = 0.92, *P* < 0.001). The risk was slightly lower for plant-based diets than for energy-dense diets (HR = 0.97, *P* = 0.025), whereas there was no significant difference for traditionally nutritious diets (HR = 0.98, *P* = 0.254). The low SES population had the highest risk (HR = 1.54, *P* < 0.001), with a 54% increased risk compared with the high SES population; the moderate SES population had a 17% increased risk, both of which were significant (HR = 1.17, *P* < 0.001). For every standard deviation increase in surrounding vegetation (NDVI), the risk was reduced by approximately 5% (HR = 0.95, *P* = 0.028).

**Table 2:** Associations of outdoor activity, dietary patterns, socioeconomic status, and the NDVI with the risk of myopia progression to high myopia

	Events/N	HR	95% CI	*P-*value
Outdoor time				
*0-2 h*	20,255/147,408			
*2 h above*	2,968/24,180	0.92	0.89 ~ 0.96	<0.001
Dietary pattern				
*Energy-dense pattern*	8,503/61,089			
*Plant-based pattern*	8,115/60,529	0.97	0.94~1.00	0.025
*Traditional nutrient-rich pattern*	6,605/49,970	0.98	0.95~1.01	0.254
SES				
*High*	7,770/63,042			
*Medium*	8,537/57,912	1.17	1.13~1.21	<0.001
*Low*	9,616/50,634	1.54	1.46~1.61	<0.001
NDVI				
*Per std*		0.95	0.91 ~ 1.00	0.028

### Sensitivity analysis

To minimise confounding factors and enhance the robustness of the statistical results, we conducted a sensitivity analysis of the multifactor regression (Table S5 in the **Online Supplementary Document**). Outdoor time >2 hours (HR = 0.93, *P* < 0.01) and low SES (HR = 1.49, *P* < 0.01) remained strong risk factors. The results of the plant-based pattern-adjusted sensitivity analysis were more significant (HR = 0.94, *P* < 0.01). NDVI-adjusted sensitivity analysis also demonstrated a significant association (HR = 0.93, *P* < 0.01). The traditional nutrient-rich pattern was statistically significant after sensitivity analysis adjustment (HR = 0.96, *P* = 0.05).

### Incidence of HMs with outdoor activity time, SES, and the NDVI

The stratified and interaction analyses revealed significant heterogeneity in the association between outdoor time and myopia progression (**Figure 2**, Panels A–B). A significant interaction was observed between outdoor time and NDVI (**Figure 2**, Panel B, *P* = 0.01). Children residing in high-NDVI areas (top tertile) exhibited a generally lower risk across outdoor activity categories (HR = 0.90; 95% CI = 0.85–0.95) compared with those in low-NDVI regions (HR = 0.94; 95% CI = 0.90–0.98), suggesting that greener environments may provide a protective baseline in which additional outdoor duration conferred limited incremental benefit. Conversely, SES modified the association in the opposite direction: the protective association of outdoor time was attenuated in low-SES populations (HR = 0.96; 95% CI = 0.92–1.01, *P* = 0.03) compared with their high-SES counterparts (HR = 0.89; 95% CI = 0.85–0.93).

**Figure 2:**
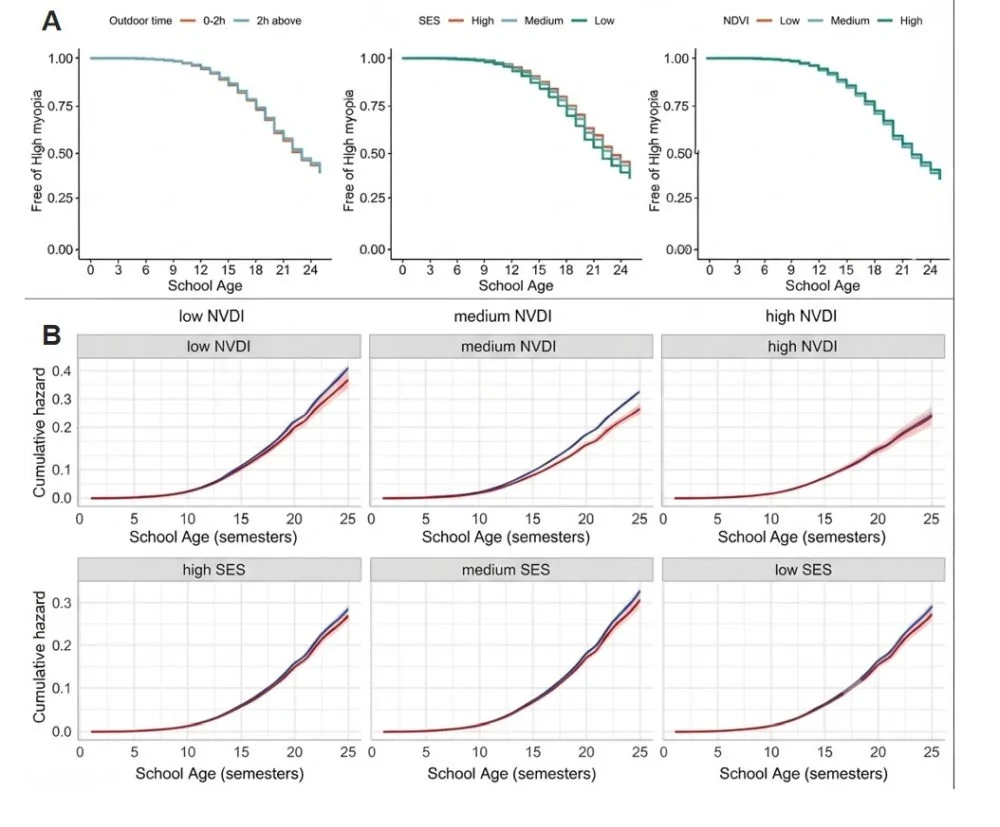
Main and interactive associations of daily outdoor activity, socioeconomic status, and neighbourhood greenness with incident high myopia over school age. Panel A. Associations between outdoor activity time, socioeconomic status (SES), and neighbourhood greenness (NDVI) and incident high myopia. Students with more than two hours of daily outdoor activity had a significantly lower risk of developing high myopia (*P* < 0.05). Higher SES and greater NDVI were both associated with a reduced incidence of high myopia. Panel B. Interaction between outdoor activity duration and neighbourhood greenness (NDVI) on high myopia risk. The association between outdoor time and risk differed across NDVI strata (*P*-interaction = 0.01). Children in high-NDVI environments generally exhibited lower risk levels, with minimal variation across outdoor categories, whereas greater differentiation by outdoor duration was observed in low-NDVI environments. NDVI – Normalized Difference Vegetation Index, SES – socioeconomic status.

Subgroup analyses further revealed variations in effects (**Figure 3**). The protective effect was most robust in younger children aged 6–12 years (HR = 0.88; 95% CI = 0.82–0.95, *P* < 0.001) but diminished during adolescence (13–18 years: HR = 0.96; 95% CI = 0.92–1.01, *P* = 0.03). Stratification by baseline vision status revealed more substantial effects among nonmyopic children (HR = 0.85; 95% CI = 0.80–0.91) than among those with mild myopia (HR = 0.95; 95% CI = 0.90–1.00, *P* = 0.01). Marginally greater efficacy was noted in rural *vs.* urban settings (HR = 0.89 *vs.* 0.92, *P* < 0.01). The sex-stratified results showed HRs of 0.90 in males and 0.94 in females (*P* > 0.05).

**Figure 3:**
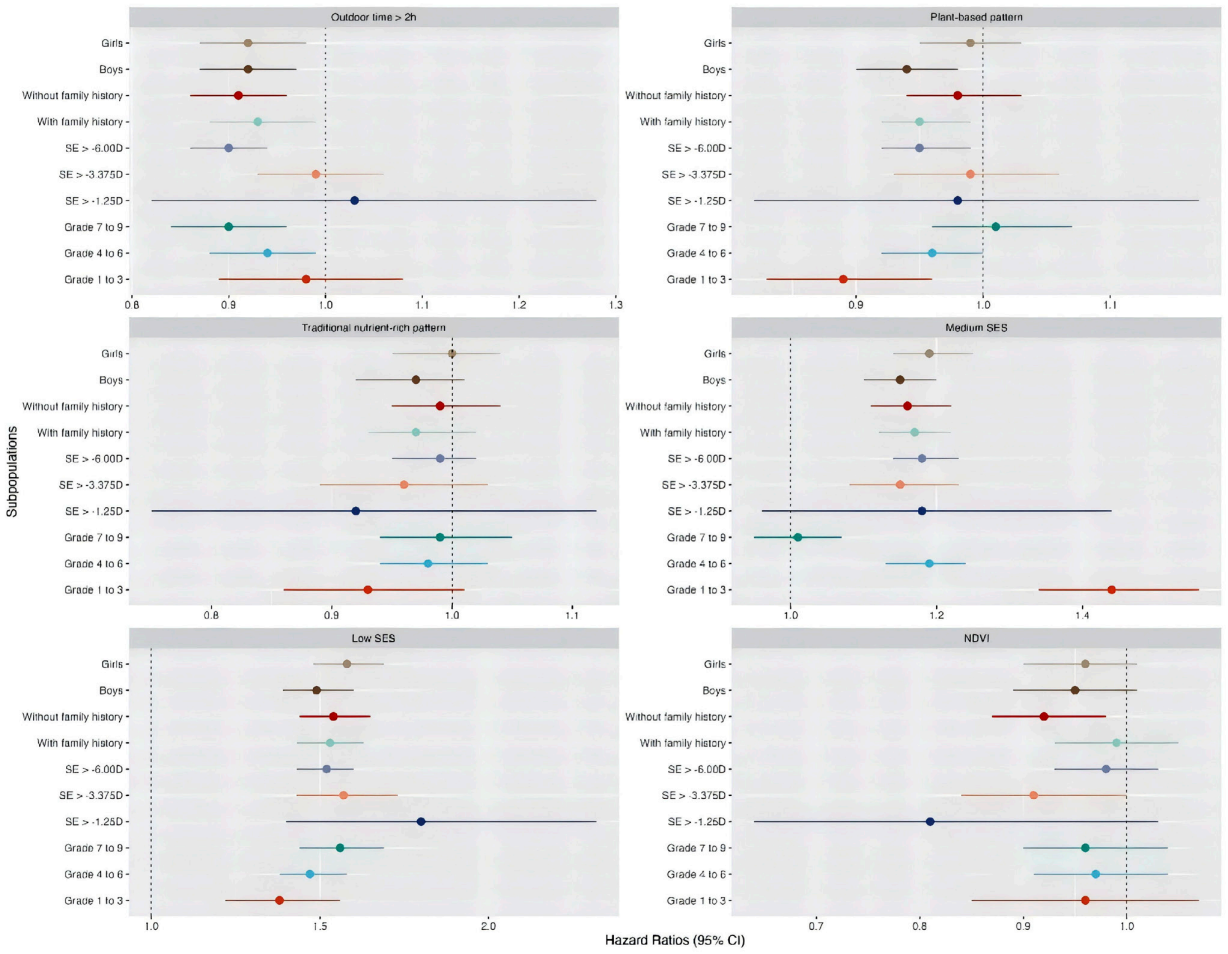
Risk factor analysis in different subgroups. Subgroup analysis of risk factors for high myopia progression. Stratified models by age, baseline refraction, and residential context revealed that outdoor time had the most potent protective effect on children aged 6–12 years and in high-NDVI regions.

## DISCUSSION

This large-scale, multisite cohort study investigated multilevel determinants of HM progression among 204,238 school-aged children in Tianjin over a critical 4-year period. By integrating socioecological contexts (area-level SES, neighbourhood greenness), behavioural factors (outdoor activity, dietary patterns), and individual characteristics, we provide robust evidence on the complex trajectories leading to HM. The significant incidence of HMs (13.2%) highlights the need to identify modifiable risk factors within ecological and behavioural frameworks to inform targeted prevention strategies.

Our findings reveal a pronounced socioeconomic gradient in HM risk, with children in low-SES regions facing a 54% increased hazard compared with their high-SES counterparts. This association persisted after rigorous sensitivity analysis (HR = 1.49, *P* < 0.01), suggesting that SES is a fundamental upstream determinant that transcends individual risk profiles. A meta-analysis revealed a strong positive correlation between the incidence and rate of myopia and socioeconomic status in East Asia [26]. Pan *et al*. analysed cross-sectional population data from China in 2014 concerning the economic environment and reported a correlation between vision loss and the economy. Our study of real-world data and our focus on the economic dynamics of myopia and HM yielded similar results [13]. Critically, **Figure 1** confirms this spatially: schools in low-SES districts cluster in densely urbanised zones with minimal green cover (pale dots), contrasting sharply with high-SES/high-NDVI clusters. This likely reflects compounded disadvantages in healthcare access (*e.g.* delayed myopia diagnosis/management), educational pressures, environmental quality, and reduced adoption of preventive measures in resource-poor settings. These results extend prior ecological theories by quantitatively linking regional SES metrics (GDP allocation, education/health investment) to a critical clinical endpoint, HM progression, and call for equity-focused public health policies.

Critically, the effectiveness of behavioural interventions appeared to depend on socioeconomic context. The protective effect of outdoor activity was significantly attenuated in low-SES areas. Previously, many studies have investigated the relationship between outdoor exercise and the time of myopia onset and reported that outdoor exercise is correlated with the onset of myopia in a dose-dependent manner [27,28]. Sedentary and close eye use also increases the likelihood of myopia [29,30]. Our study not only focuses on the role of the outdoors in the progression from myopia to HM but also incorporates interaction effects across economic regions. This suggests that structural factors such as the availability of recreational space and academic demands may contribute, although these pathways were not directly measured. Consequently, interventions that promote ‘more outdoor time’ may inadvertently widen disparities unless paired with investments in neighbourhood infrastructure and socioeconomic support.

The protective role of sustained outdoor activity (>2 hours/d; HR = 0.92) aligns with established biological mechanisms (dopamine release, reduced accommodative stress). Our interaction analysis suggested that neighbourhood greenness modified the association between outdoor activity and high myopia risk (*P* = 0.01). Children living in greener environments exhibited generally lower risk levels regardless of outdoor duration, whereas in less green environments, longer outdoor exposure appeared to provide a more pronounced protective association. This finding may indicate that environmental context influences the marginal benefit of behavioural interventions. In response to the occurrence and risk factors of myopia in children, previous studies on spatial frequency and greening have tentatively shown a protective effect between the two [5,6,31]. This suggests that NDVI may not merely serve as a proxy for outdoor exposure but may represent broader environmental characteristics, such as ambient light conditions, opportunities for incidental outdoor activity, or reduced visual environmental stress.

Several biological and behavioural pathways may explain the observed protective associations of outdoor exposure and greener environments with high myopia progression. Increased light intensity outdoors is known to stimulate retinal dopamine release, which acts as a key neuromodulator inhibiting axial elongation through dopamine D2 receptor-mediated signalling pathways [32]. Experimental animal studies and human epidemiological evidence consistently support the role of bright-light exposure in slowing ocular growth [33,34]. In addition, outdoor activity may reduce sustained near-work-induced accommodative stress and peripheral hyperopic defocus, both of which have been implicated in scleral remodelling and axial elongation [35–37]. Greener environments may further contribute through indirect mechanisms, including promoting physical activity, reducing psychological stress, and improving overall well-being [31]. Chronic stress has been hypothesised to influence ocular growth *via* autonomic and hormonal pathways, including cortisol-mediated remodelling of the scleral extracellular matrix [38,39]. Therefore, the combined effects of higher ambient light exposure, increased physical activity, and reduced environmental stress may jointly contribute to the protective associations observed in children living in greener environments.

The stronger protective effect of outdoor activity in younger children (6–12 years: HR = 0.88 *vs.* adolescents: HR = 0.96, *P* = 0.03) highlights a critical window for intervention before peak growth velocity and myopic progression accelerate. Additionally, while the dietary effects were modest, a plant-based pattern was associated with a small but significant reduction in HM risk (HR = 0.97), which strengthened upon sensitivity adjustment (HR = 0.94, *P* < 0.01). Previous studies revealed a correlation between the occurrence of myopia in American children and carbohydrate diets and sugary drinks [15,40]. Two studies in China came to a similar conclusion that sugary drinks and being overweight are both risk factors for myopia. They were cross-sectional studies, and again, they did not examine the mediating role of diet in the progression of myopia to HM in a myopic population [21,41]. Plant-based dietary patterns were associated with a moderate reduction in the risk of HMs (HR = 0.97), which remained robust after sensitivity adjustment (HR = 0.94). Traditional nutrient-rich patterns showed a borderline benefit only after full adjustment (HR = 0.96, *P* = 0.05), suggesting interactions with unadjusted covariates. These findings warrant investigating diet-scleral remodelling pathways.

Previous studies on the relationship between risk factors for myopia and individual eye use in Hangzhou children have shown that myopia development is associated with various eye-use-related factors; however, these studies have been hindered by many confounding factors [42]. Our previous study revealed a lower risk of myopia in rural areas but did not focus on the dynamics of progression to HM [23]. Hence, we performed subgroup clustering across different dimensions, revealing key risk factor differences associated with the development of HMs. First, when stratified by age subgroup, outdoor activity was significantly more protective in children (HR = 0.88) and adolescents (HR = 0.96, *P* = 0.03) aged 6–12 years, suggesting that early intervention during a critical developmental window maximises the benefits. In addition, among baseline refractive status subgroups, children with mild myopia (HR = 0.85) were significantly better protected against outdoor exposure than those with moderate myopia (HR = 0.95, *P* = 0.01), highlighting differences in biological susceptibility across myopia stages. Additionally, we analysed different regions, demonstrating a rural advantage: despite differences in overall exposure, rural participants had slightly greater outdoor benefits (HR = 0.89) than urban participants (HR = 0.92), reflecting the influence of unmeasured environmental factors (*e.g.* less stressful work environments near their homes).

This study inevitably has several limitations. First, area-level SES and NDVI assignment limit granularity in individual exposure assessment, and changes in behavioural patterns over time were not modelled, which may attenuate observed associations. Second, outdoor time and dietary data rely on parent-child reports, which are vulnerable to recall and social desirability bias. Seasonal variation in daylight exposure and air quality may influence outdoor behaviour and ocular growth; future studies incorporating season-specific exposure measurements are warranted. Third, geographic scope may limit generalisability to rural China or other cultural contexts with differing lifestyles and SES structures. Such nondifferential misclassification would likely bias estimates toward the null rather than to produce spurious associations. A deeper interrogation of how SES mediates risk was infeasible. Future longitudinal studies are recommended to incorporate objective measures (light sensors, retracking) and targeted mechanistic research on plant-based diet components and structural inequities. Additionally, outdoor activity and dietary information were derived from parent-reported questionnaires and are therefore susceptible to recall bias and social desirability bias, which may introduce nondifferential exposure misclassification.

## CONCLUSIONS

This study revealed that a multilevel interaction of factors drove children's HM progression: children in low-SES regions had a significant 54% increased risk of HMs, highlighting the dominant role of structural inequalities. At the same time, two hours of outdoor activity per day lowered the risk; its protective effect was significantly moderated by NDVI relative to SES; its protective association was significantly modified by environmental and socioeconomic context; children in greener areas exhibited generally lower risk levels, whereas low-SES regions substantially attenuated the benefits. The core window of intervention exists for nonmyopic children aged 6–12 years, calling for integrated policies that reduce geographic health disparities by targeting resources, increasing green space in schools in conjunction with urban planning, and promoting early behavioural interventions for key age groups to break the negative socioenvironmental-behavioural cycle of myopia progression.

## Additional material


Online Supplementary Document

## Data Availability

**Data availability:** The data that support the findings of this study are available from the corresponding author upon reasonable request.
