# Supplementary material for: Influence of environmental, economic, dietary, and behavioural factors on high myopia onset in children: a 4-year large-scale cohort study
Source: J Glob Health. 2026 Oct 2;16:04298. doi: 10.7189/jogh.16.04298 (PMC13634204; doi:10.7189/jogh.16.04298)
Supplement: Supplementary file 1 [file jogh-16-04298-s001.pdf]

Supplement to: Wang Z, Yu J, Song D, Wang J, Yan H, Du B, Ruihua W. Influence of environmental, economic, dietary, and behavioural factors on high myopia onset in children: a 4-year large-scale cohort study. J Glob Health. 2026;16:04298.

Table S1. Description and equipment related to specific ophthalmologic examinations in this study. (Page 2)

Table S2. The description of questions used in this study was derived from the TCARE genetic, behavioral, and dietary questionnaire. (Page 3-5)

Figure S1. Dietary data from questionnaire sources were classified into three nutritional patterns based on factor analysis. (Page 6)

Table S3. Guidance for deriving socio-economic conditions at the district level. (Page 7)

Table S4. Description of variables used to create the area-level socio-economic status indicator. (Page 8)

Figure S2. Three-level classification of ESE in different areas of Tianjin based on principal components. (Page 9)

Table S5: Sensitivity analysis: robustness validation of outdoor activities, dietary patterns, socioeconomic status, and NDVI on the risk of myopia progression. (Page 10)

Table S1: Description and equipment related to specific ophthalmologic examinations in this study

Participants were directed to discontinue optical correction before undergoing cycloplegic refraction and monocular uncorrected visual acuity (UCVA) assessment. Certified optometrists who had completed uniform training protocols administered all ocular examinations, performing bilateral refractive measurements and vision testing for each study subject. UCVA assessment was conducted with a logarithmic E-chart at a 5-meter testing distance. Right-eye (OD) measurements preceded left-eye (OS) evaluations using an opaque ocular occlud, following a sequential testing protocol with 5- 10s intermission between ocular examinations to mitigate inter-eye adaptation effects. Testing sequences incorporated brief adaptation intervals to minimize lingering occlusion effects. Duplicate measurements under corrected and uncorrected conditions were obtained for spectacle users.

Refractive status was quantified using the Tianle RM-9600 autorefractor (Shanghai, China) through triplicate noncycloplegic measurements per eye, with subsequent averaging. Repeat assessments were mandated when undermeasurement discrepancies exceeded 0.50D in any axis. Spherical equivalent (SE) derivation followed standard arithmetic computation of sphere plus half cylindrical power.

Table S2. The description of questions used in this study was derived from the TCARE genetic, behavioral, and dietary questionnaire.

Section	Field Label (English)	Field Type	Options/Input Format
**I. Basic Info**	Student Name	Text Input	Free text
	Gender	Single Choice	○ Male<br>○ Female
	Date of Birth	Text Input	
	School Name	Text Input	
	Grade & Class	Text Input	Grade: ______ Year<br>Class: ______ Class
**II. Biometrics**	Height (cm)	Numeric Input	cm
	Weight (kg)	Numeric Input	kg
**III. Ocular History**	Eye Surgery History	Single Choice	○ Yes<br>○ No
	Parental Highest Education	Single Choice	○ Postgraduate<br>○ Bachelor's<br>○ College<br>○ Technical Secondary<br>○ High School<br>○ Below High School
**IV. Genetic Factors**	Parental Myopia History	Single Choice	○ Yes (Specify: _______________)<br>○ No
	Parental High Myopia History (-6.00D+)	Single Choice	○ Yes (Specify: _______________)<br>○ No
**V. Sleep Patterns**	Bedtime	Time Input	HH:MM
	Wake-up Time	Time Input	HH:MM
**VI. Activity Profile**	Daily Outdoor Time	Single Choice	○ <1h<br>○ 1-2h

Section	Field Label (English)	Field Type	Options/Input Format
			○ 2-3h<br>○ >3h
	Weekly Exercise Frequency	Single Choice	○ 1 day/week<br>○ 2 days<br>○ 3 days<br>○ 4 days<br>○ 5 days<br>○ 6 days<br>○ 7 days
**VII. Vision Correction**	Current Eyewear Use	Single Choice	○ Yes<br>○ No
**VIII. Screen Time**	Recreational Screen Use (Non-study)	Single Choice	○ <1h<br>○ 1-2h<br>○ 2-3h<br>○ 3-4h<br>○ 4-5h<br>○ >5h
	Educational Screen Use	Single Choice	Same options as above
**IX. Reading Habits**	Paper Material Reading	Single Choice	Same duration options
**X. Dietary Patterns**	Whole Grains Intake	Single Choice	○ Rarely<br>○ <1/week<br>○ 2-3/week<br>○ 4-6/week<br>○ 1/day<br>○ ≥2/day
	Seafood Consumption	Single Choice	○ Rarely<br>○ <1/week<br>○ 2-3/week<br>○ 4-6/week<br>○ 1/day<br>○ ≥2/day
	Fresh Fruit Intake	Single Choice	○ Rarely<br>○ <1/week

Section	Field Label (English)	Field Type	Options/Input Format
			○ 2-3/week<br>○ 4-6/week<br>○ 1/day<br>○ ≥2/day
	Fresh Vegetable Intake	Single Choice	○ Rarely<br>○ <1/week<br>○ 2-3/week<br>○ 4-6/week<br>○ 1/day<br>○ ≥2/day
	Snacks/Sweets Intake	Single Choice	○ Rarely<br>○ <1/week<br>○ 2-3/week<br>○ 4-6/week<br>○ 1/day<br>○ ≥2/day
	Fried/Barbecued Foods Intake	Single Choice	○ Rarely<br>○ <1/week<br>○ 2-3/week<br>○ 4-6/week<br>○ 1/day<br>○ ≥2/day
	Sugary Beverages Intake	Single Choice	○ Rarely<br>○ <1/week<br>○ 2-3/week<br>○ 4-6/week<br>○ 1/day<br>○ ≥2/day
	Daily Salt Intake	Single Choice	○ <4g (Low)<br>○ 4-6g (Normal)<br>○ >6g (High)

Figure S1. Dietary data from questionnaire sources were classified into three nutritional patterns based on factor analysis.

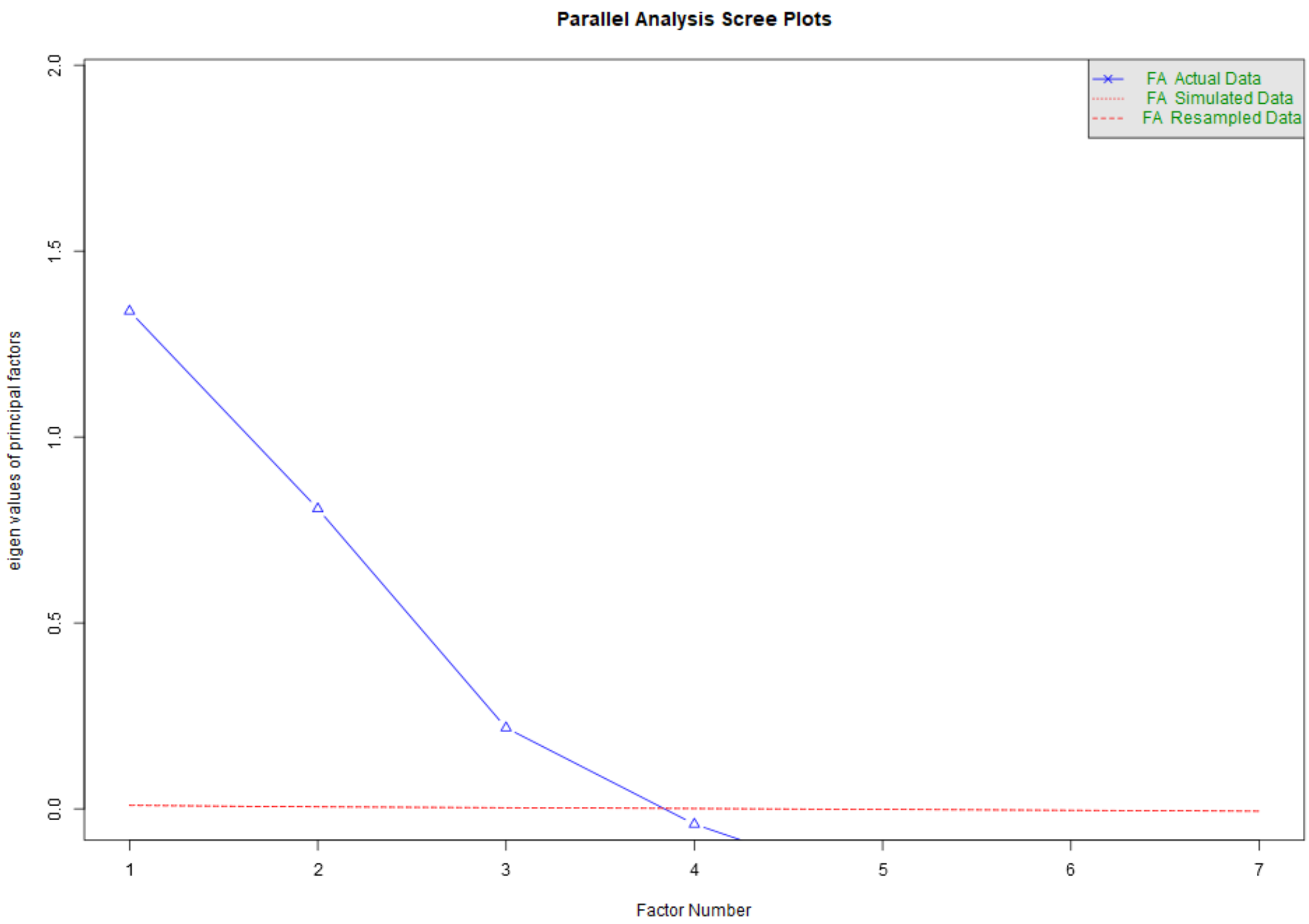

Table S3. Guidance for deriving socio-economic conditions at the district level.
This study's area-level socioeconomic status (SES) was developed via a predictive model integrating economic, educational, and healthcare indicators. Data were obtained from the 2020 annual report of the Tianjin Statistical Bureau, published in the 2021 Tianjin Statistical Yearbook. District-level metrics included population, gross domestic product (GDP) (in 100 million yuan), educational budgetary expenditures (in 100 million yuan), health expenditure (in 100 million yuan), licensed (assistant) physicians per 1,000 residents, registered nurses per 1,000 residents, and hospital beds per 1,000 residents. Derived metrics included regional GDP per capita, educational expenditure-to-GDP ratio, health expenditure-to-GDP ratio, healthcare professionals per 1,000 residents (physicians and nurses combined), and hospital beds per 1,000 residents. These variables comprised the SES composite indicator.

Standardized Z-scores were computed as:

$$Z'_{ij} = (x_{ij} - \bar{X}) / s_j \ (i = 1,2, ..., n; j = 1,2, ..., m)$$

For principal component analysis (PCA). A correlation matrix derived from standardized data underwent eigenvalue decomposition, with the first two principal components selected based on eigenvalue magnitudes to capture maximal variance. The derived composite *f*-score was calculated via a linear combination.

$$f = c_1Z_1 + c_2Z_2 + ... + c_pZ_p$$

Districts were stratified into high- (Heping, Hexi, Hebei, Hongqiao, Nankai), middle- (Binhai New Area, Jinnan, Beichen, Hedong, Baodi, Xiqing), and low-level SES (Dongli, Ninghe, Jizhou, Jinghai, Wuqing) tiers based on ranked values. Raw data and standardized metrics are detailed in Tables S2.1 and S2.2, respectively.

Table S4. Description of variables used to create the area-level socio-economic status indicator.

Area	Hci	Beds	educ	PerGDP	City
Heping	210	4751	0.31710143	23.34274407	0.985101931
Hedong	217	3603	0.250473614	4.001712396	0.932183878
Hexi	321	9150	0.267806608	12.0363102	0.953964714
Nankai	334	9374	0.288227033	7.031989056	0.914821656
Hebei	211	4026	0.305620045	5.640563737	0.669569547
Hongqiao	140	3645	0.253996941	3.838153885	0.940016789
Dongli	255	1807	0.131426336	7.588759276	0.580946498
Xiqing	270	3076	0.15674724	6.866293437	0.551548433
Jinnan	220	3843	0.099515751	5.809427587	0.653391087
Beichen	217	4786	0.179869666	6.861770523	0.542345782
Wuqing	710	4043	0.181042741	7.083446775	0.615363322
Baodi	424	2698	0.229324923	4.9054892	0.581328628
Binhai	758	8220	0.079178663	28.39965745	0.542929273
Ninghe	319	1415	0.257181281	7.912419449	0.56157107
Jinghai	489	1959	0.198484826	5.644398099	0.497255651
Jizhou	741	1985	0.278549595	2.823361152	0.634692864

Figure S2. Three-level classification of ESE in different areas of Tianjin based on principal components.

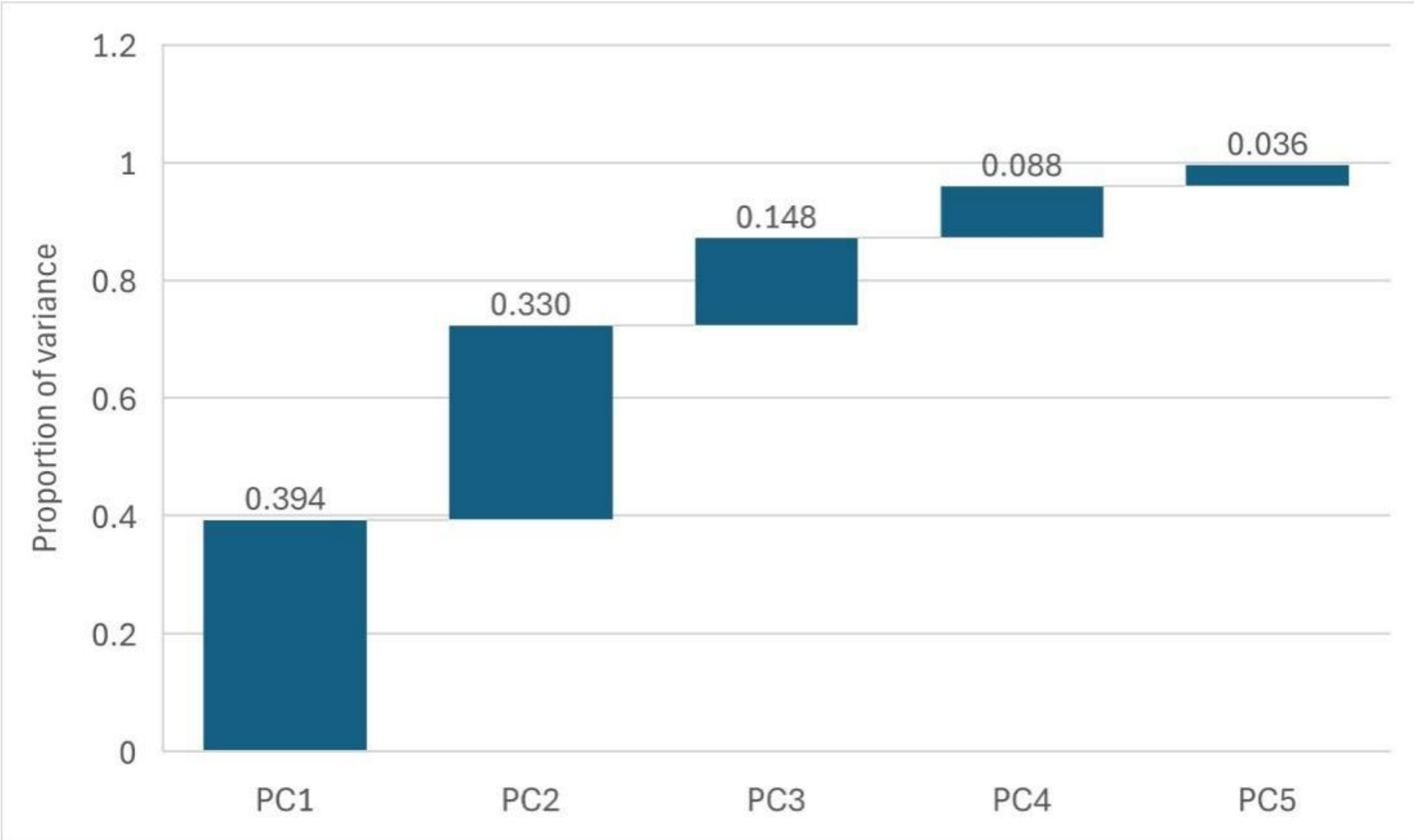

Table S5: Sensitivity analysis: robustness validation of outdoor activities, dietary patterns, socioeconomic status, and NDVI on the risk of myopia progression

	HR	95% CI	*P*
**Outdoor time**			
0-2h			
2h above	0.93	0.89 ~ 0.98	<0.001
**Dietary pattern**			
Energy-dense pattern			
Plant-based pattern	0.94	0.91□ 0.98	0.004
Traditional nutrient-rich pattern	0.96	0.93□ 1.00	0.051
**SES**			
High			
Medium	1.19	1.14□ 1.23	<0.001
Low	1.49	1.40□ 1.57	<0.001
**NDVI**			
Per std	0.93	0.88~0.98	<0.001
